# Neutrophil-to-lymphocyte ratio and monocyte-to-lymphocyte ratio predict length of hospital stay in myocarditis

**DOI:** 10.1038/s41598-021-97678-6

**Published:** 2021-09-13

**Authors:** Moritz Mirna, Lukas Schmutzler, Albert Topf, Uta C. Hoppe, Michael Lichtenauer

**Affiliations:** Department of Internal Medicine II, Division of Cardiology, Universitätsklinikum Der Paracelsus Medizinischen Universität, Müllner Hauptstraße 48, 5020 Salzburg, Austria

**Keywords:** Cardiology, Cardiovascular diseases, Heart failure

## Abstract

Neutrophil-to-lymphocyte ratio (NLR) and monocyte-to-lymphocyte ratio (MLR) are associated with the severity of various diseases. The aim of this study was to demonstrate the relationship of NLR and MLR with the severity of myocarditis. 202 consecutive patients with myocarditis were retrospectively enrolled in this study. Laboratory parameters and clinical data were extracted from hospital records and discharge letters. Median NLR was 2.48 (IQR 1.55–4.58) and median MLR was 0.42 (IQR 0.39–0.58). NLR and MLR correlated with HF, CRP and leukocyte count, MLR further correlated inversely with LV systolic function (rs = − 0.379, *p* = 0.030). Both ratios correlated better with length of hospital stay (NLR: rs = 0.435, *p* = 0.003; MLR: rs = 0.534, *p* < 0.0001) than CRP, leukocyte count, IL-6 or procalcitonin. AUCs for the prediction of prolonged hospital stay (NLR = 0.75, MLR = 0.80), and optimal cut-offs therefor were calculated. Patients who had in-hospital complications showed a higher NLR, however, this remained statistically insignificant. NLR and MLR correlated with the length of stay, as well as with several clinical and laboratory parameters in patients with myocarditis. Since white blood cell differentials are relatively easy and fast to perform, both ratios could facilitate further risk stratification in affected patients.

## Introduction

The term ‘myocarditis’ refers to inflammatory processes within the myocardium, which are most frequently a result of infections with cardiotropic viruses such as coxsackie B virus, parvovirus B19 or adenoviruses^1,2^. Viral infection and lysis of infected cardiomyocytes lead to an activation of the innate and adaptive immune response, which commonly results in an attenuation of inflammation and hence recovery. Infrequently, however, the inflammatory response persists, leading to a persistent decline of left ventricular systolic function or fatal adverse outcomes, as cardiomyocytes are unabatedly being destroyed^2,3^.

While inflammatory responses are generally beneficial processes to oppose impeding pathogens, severe inflammation and exaggerated immunological responses can result in prolonged hospital stay, end-organ damage and adverse outcomes for affected patients. To identify patients at risk is thus crucial for enhanced surveillance and early targeted therapy to improve outcomes^4–7^.

In 2001, Zahorec investigated white blood cell differentials in 90 oncological patients on the intensive care unit, and reported that critically ill patients with severe sepsis or septic shock showed marked neutrophilia and lymphopenia. Furthermore, the ratio of neutrophils to lymphocytes correlated well with the severity of clinical courses, as assessed by SOFA and APACHE II scores^8^. In the following years, an ever increasing number of studies showed that neutrophil-to-lymphocyte ratio (NLR) could adequately predict severity in several disease entities, for example in community-acquired pneumonia, sepsis, hepatocellular carcinoma and even COVID-19^9–12^. Similarly, monocyte-to-lymphocyte ratio (MLR) was previously associated with adverse outcomes in different forms of cancer^13,14^, but also in patients with non-ST-segment elevation myocardial infarction^15^. In contrast to NLR and MLR, evidence on eosinophil-to-lymphocyte ratio (ELR) and basophil-to-lymphocyte ratio (BLR) is comparatively scarce, and previous studies investigated these ratios primarily in the context of nasal polyposis and allergic rhinitis^16–18^.

While NLR and MLR have been described in a large number of disease entities previously, there is, to the best of our knowledge, currently no evidence available regarding their predictive potential in patients diagnosed with myocarditis. Hence, we aimed to investigate NLR, MLR, ELR and BLR in myocarditis in our study. Since these parameters require solely an inexpensive and expeditious white blood cell differential, an association with clinical and outcome parameters could facilitate early risk stratification and thus clinical management in affected patients.

## Results

### Baseline characteristics

In total, 202 patients with myocarditis were enrolled in this study. The median age of all patients was 36 years (IQR 25–50) and the majority of patients was male (78.7%, n = 159). Median heart frequency (HF) was 75 bpm (IQR 62–88) and median oxygen saturation was 98% (IQR 97–98, see Table 1).

**Table 1:** Baseline characteristics and comorbidities.

	Median	IQR
Age (years)	36	25–50
Systolic blood pressure (mmHg)	130	120–147
Diastolic blood pressure (mmHg)	80	70–88
Heart frequency (bpm)	75	62–88
Peripheral oxygen saturation (%)	98	97–98
Temperature (°C)	36.4	36.1–36.9

	%	n
Male sex	78.7	159
Diabetes mellitus	5.5	11
Hyperlipidemia	16.4	33
Obesity (BMI > 30 kg/m^2^)	13.4	27
Arterial hypertension	21.9	44
History of smoking	32.8	66
Chronic infectious disease	1.5	3
Autoimmune disorder	7.9	16
Immunosuppressive therapy	4.5	9
Active malignancy	3.5	7

Regarding comorbidities, autoimmune disorders were present in 7.9% (n = 16), active malignancies in 3.5% (n = 7) and chronic infectious diseases in 1.5% (n = 3, see Table 1). Nine patients received immunosuppressive therapy (4.5%), of whom five were treated with corticosteroids, with a median hydrocortisone equivalent dose^19^ of 20 mg (IQR 9.5–35).

### Laboratory findings and transthoracic echocardiography (TTE)

At the time of enrollment, the median plasma level of C-reactive protein (CRP) was 266.67 nmol/l (IQR 57.14–733.33), the median leukocyte count was 8.75 G/l (IQR 7.02–11.31) and the median concentration of interleukin-6 (IL-6) was 27.7 pg/ml (IQR 7.2–69.6). Regarding cardiac enzymes, the median plasma level of high sensitivity troponin (hsTnT) was measured at 180 ng/l (IQR 17–498), whereas pro brain natriuretic peptide (pBNP) was at 285 ng/l (IQR 103–858; see Table 2).

**Table 2:** Laboratory findings.

	Median	IQR
**Laboratory findings**		
Creatinine (µmol/l)	79.60	70.74–88.42
eGFR (ml/min/1.73m^2^)	70	70–70
C-reactive protein (CRP) (nmol/l)	266.67	57.14–733.33
Bilirubin (µmol/l)	10.26	6.84–13.68
Creatinine kinase (CK) (IU/l)	194	91–378
CK-MB (%)	10.4	8.2–13.6
high sensitivity troponin (hsTnT) (ng/l)	180	17–498
pro brain natriuretic peptide (pBNP) (ng/l)	285	103–858
Prothrombin time (%)	98	89–105
Interleukin 6 (pg/ml)	27.7	7.2–69.6
Procalcitonin (µg/l)	0.20	0.10–0.33
Hemoglobin (mmol/l)	8.94	8.19–9.62
Leukocyte count (G/l)	8.75	7.02–11.31
Thrombocyte count (G/l)	221	183–261
Neutrophils (G/l)	4.95	3.58–7.13
Lymphocytes (G/l)	1.68	1.29–2.20
Monocytes (G/l)	0.65	0.48–1.17
Eosinophils (G/l)	0.07	0.04–0.13
Basophils (G/l)	0.02	0.02–0.04
Neutrophil-to-lymphocyte ratio	2.48	1.55–4.58
Eosinophil-to-lymphocyte ratio	0.07	0.03–0.16
Basophil-to-lymphocyte ratio	0.02	0.00–0.03
Monocyte-to-lymphocyte ratio	0.42	0.30–0.58
**Transthoracic echocardiography**		
Ejection Fraction (%)	55	50–60
LV end-diastolic diameter (mm)	47	44–50
Interv. septum thickness (mm)	11	9–12

Abbreviations: IQR = interquartile range, eGFR = estimated glomerular filtration rate, CK-MB = creatinine kinase muscle-brain type.

Median neutrophil-to-lymphocyte ratio (NLR) was measured at 2.48 (IQR 1.55–4.58), whereas monocyte-to-lymphocyte ratio (MLR) was at 0.42 (IQR 0.30–0.58). Eosinophil-to-lymphocyte ratio (ELR) was 0.07 (IQR 0.03–0.016), whereas basophil-to-lymphocyte ratio (BLR) was 0.02 (IQR 0.00–0.03; see Table 2). We found no statistically significant differences in any of the four ratios investigated between patients with autoimmune disorders, active malignancies or chronic infectious diseases when compared to the rest of the cohort.

Patients who met the combined endpoint of in-hospital complications showed a trend towards higher values of NLR and ELR, which, however, remained statistically insignificant (NLR: median 3.59 (IQR 1.60–10.13) vs. 2.35 (IQR 1.55–4.15), *p* = 0.211; ELR: median 0.09 (IQR 0.01–0.17) vs. 0.07 (IQR 0.04–0.16), p = 0.823).

Concerning the measurements of TTE, median left ventricular (LV) systolic function was at 55% (IQR 50–60), the median LV end-diastolic diameter at 47 mm (IQR 44–50) and the median interventricular septum thickness at 11 mm (IQR 9–12; see Table 2).

### Diagnostic procedures, complications and outcome

The median length of hospital stay was 5 days (IQR 4–7). Cardiac MRI was conducted in 166 patients (83.4%), endomyocardial biopsy in 17 patients (8.6%), coronary angiography in 95 patients (47.0%), coronary computed tomography in 27 patients (13.4%) and myocardial scintigraphy in 3 patients (1.5%).

In-hospital complications occurred in 12.4% of all patients (n = 25). As such, relevant arrhythmias were present in 10.4% (n = 21), cardiogenic shock in 2.0% (n = 4) and mechanical ventilation was necessary in 1.5% (n = 3). In-hospital-mortality was 0% (n = 0), whereas all-cause-mortality within 2 years was 2.3% (n = 3).

### Correlation analysis

Of the four ratios investigated, NLR and MLR correlated most frequently with clinical and laboratory parameters. NLR showed a significant positive correlation with HF (rs = 0.350, *p* = 0.034), CRP (rs = 0.414, *p* = 0.006) and leukocyte count (rs = 0.399, *p* = 0.009), whereas MLR correlated positively with HF (rs = 0.370, *p* = 0.024), CRP (rs = 0.341, *p* = 0.025) and IL-6 (rs = 0.900, *p* = 0.037). Furthermore, MLR showed a significant inverse correlation with LV systolic function (rs = − 0.379, *p* = 0.030).

Both NLR and MLR correlated better with the length of hospital stay (NLR: rs = 0.435, *p* = 0.003; MLR: rs = 0.534, *p* < 0.0001) than established inflammatory biomarkers (CRP: rs = 0.271, *p* < 0.0001; leukocyte count: rs = 0.292, *p* < 0.0001; IL-6: 0.396, *p* = 0.161; procalcitonin: − 0.023, *p* = 0.911; see Table 3).

**Table 3:** Correlation analysis of ratios with clinical and laboratory parameters.

		Length of stay	Syst. BP	Temp	Oxygen saturation	Heart frequency	EF	LVEDD	IVSD	Creatinine	eGFR	CRP	Bilirubin	CK
		(days)	(mmHg)	(°C)	(%)	(bpm)	(%)	(mm)	(mm)	(µmol/l)	(ml/min/1.73m^2^)	(nmol/l)	(µmol/l)	(IU/l)
NLR	rs	0.435	− 0.110	− 0.091	− 0.381	0.350	− 0.304	0.071	− 0.136	− 0.073	− 0.061	0.414	0.090	− 0.211
	*p* Value	0.003	0.654	0.758	0.352	0.034	0.085	0.713	0.482	0.646	0.699	0.006	0.670	0.180
ELR	rs	0.008	0.381	− 0.195	− 0.872	− 0.013	− 0.081	0.240	− 0.124	− 0.030	− 0.091	− 0.045	− 0.031	− 0.142
	*p* Value	0.961	0.107	0.503	0.005	0.940	0.653	0.209	0.522	0.852	0.567	0.776	0.884	0.369
BLR	rs	− 0.227	0.039	− 0.030	− 0.414	0.094	− 0.267	0.228	− 0.242	− 0.070	0.253	0.122	0.283	0.223
	*p* Value	0.139	0.874	0.918	0.308	0.580	0.133	0.235	0.206	0.662	0.106	0.437	0.170	0.155
MLR	rs	0.534	0.101	0.270	0.344	0.370	− 0.379	− 0.135	− 0.134	0.078	− 0.145	0.341	0.319	− 0.215
	*p* Value	< 0.0001	0.680	0.351	0.404	0.024	0.030	0.484	0.487	0.623	0.360	0.025	0.120	0.171

		hsTnT	pBNP	PTT	Hemoblobin	Leukocyte count	Thrombocyte count	IL-6	Procalcitonin					
		(ng/l)	(ng/l)	(%)	(mmol/l)	(G/l)	(G/l)	(pg/ml)	(µg/l)					
NLR	rs	− 0.067	0.221	− 0.152	− 0.201	0.399	0.096	0.100	0.479					
	*p* Value	0.679	0.289	0.361	0.202	0.009	0.546	0.873	0.230					
ELR	rs	− 0.144	0.193	0.109	− 0.192	0.046	0.031	− 0.500	0.126					
	*p* Value	0.370	0.355	0.516	0.224	0.772	0.845	0.391	0.767					
BLR	rs	0.087	0.042	− 0.031	− 0.144	− 0.092	− 0.095	− 0.900	0.319					
	*p* Value	0.589	0.843	0.855	0.363	0.563	0.550	0.037	0.441					
MLR	rs	− 0.095	− 0.144	− 0.219	0.123	0.292	− 0.118	0.900	− 0.295					
	*p* Value	0.554	0.493	0.187	0.439	0.061	0.456	0.037	0.479					

Abbreviations: NLR = neutrophil-to-lymphocyte ratio, ELR = eosinophil-to-lymphocyte ratio, BLR = basophil-to-lymphocyte ratio, MLR = monocyte-to-lymphocyte ratio, BP = blood pressure, EF = ejection fraction, LVEDD = left ventricular diameter end-diastole, IVSD = interventricular septum diameter, eGFR = estimated glomerular filtration rate, CRP = C-reactive protein, CK = creatinine kinase, hsTnT = high sensitivity troponin T, pBNP = pro brain natriuretic peptide, PTT = prothrombin time, IL-6 = interleukin 6.

Of note, ELR correlated inversely with oxygen saturation (rs = − 0.872, p = 0.005), but BLR did not show any relevant correlation with laboratory, clinical or echocardiographic parameters.

### Linear regression analyses

**Figure 1:**
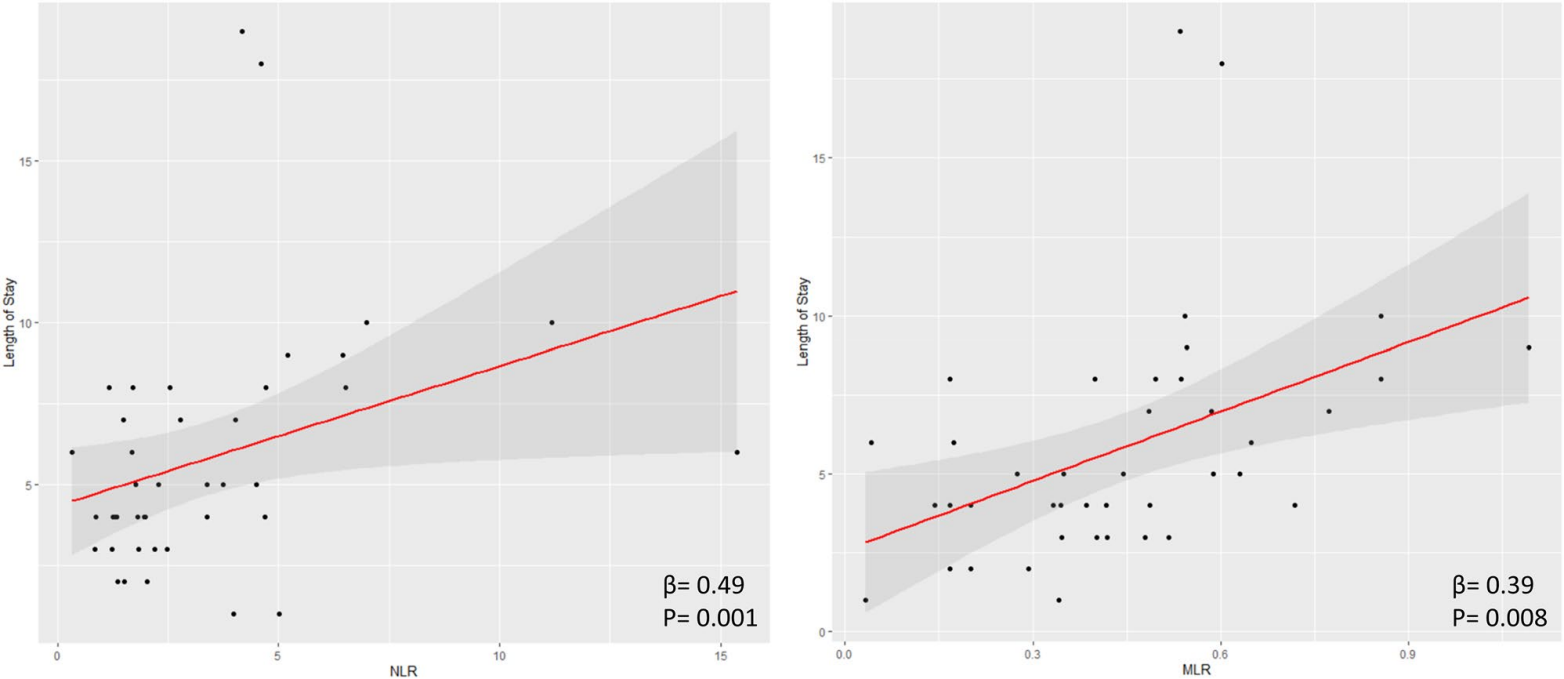
Univariate linear regression analysis of NLR and MLR with length of hospital stay. Abbreviations: NLR = neutrophil-to-lymphocyte ratio, MLR = monocyte-to-lymphocyte ratio.

In univariate linear regression analysis, NLR and MLR were significantly associated with the length of hospital stay (NLR: β = 0.49, R^2^ = 0.22, *p* = 0.001; MLR: β = 0.39, R^2^ = 0.13, *p* = 0.008, see Fig. 1). This association remained statistically significant (NLR: β = 0.18, *p* = 0.017; MLR: β = 0.24, *p* = 0.005) in a multivariate linear regression model where we corrected for parameters that have a known influence on peripheral leukocyte counts (diabetes mellitus, obesity, active smoking status, autoimmune disorder, chronic infectious disease, active malignancy, see Supplementary Table S1).

We then constructed another multiple linear regression analysis with established inflammatory parameters (CRP, leukocyte count, NLR, MLR) using the ‘backward’ method. Here, MLR achieved the best predictive ability for length of stay in patients with myocarditis (AIC = 221.18, adj. R^2^ = 0.18, F-statistic = 9.54, *p* = 0.0037, see Table 4).

**Table 4:** Multivariate linear regression analysis for the prediction of length of hospital stay using the stepwise method with backward direction.

	AIC = 108.34	adj. R^2^ = 0.12	β	VIF	*p* Value
*Constant*					*0.117*
CRP			0.014	1.628	0.943
Leukocyte count			− 0.012	1.247	0.945
NLR			0.113	2.244	0.615
MLR			0.379	1.480	0.043

	AIC = 106.35	adj. R^2^ = 0.14	β	VIF	*p* Value
*Constant*					*0.043*
Leukocyte count			0.011	1.570	0.952
NLR			0.110	2.143	0.612
MLR			0.379	1.480	0.040

	AIC = 104.35	adj. R^2^ = 0.16	β	VIF	*p* Value
*Constant*					*0.037*
NLR			0.117	1.457	0.507
MLR			0.378	1.457	0.037

	AIC = 102.83	adj. R^2^ = 0.18	β	VIF	*p* Value
*Constant*					*0.033*
MLR			0.443		0.004

Abbreviations: CRP = C-reactive protein, NLR = neutrophil-to-lymphocyte ratio, MLR = monocyte-to-lymphocyte ratio, AIC = Akaike information criterion, adj. = adjusted, VIF = variance inflation factor.

**Figure 2:**
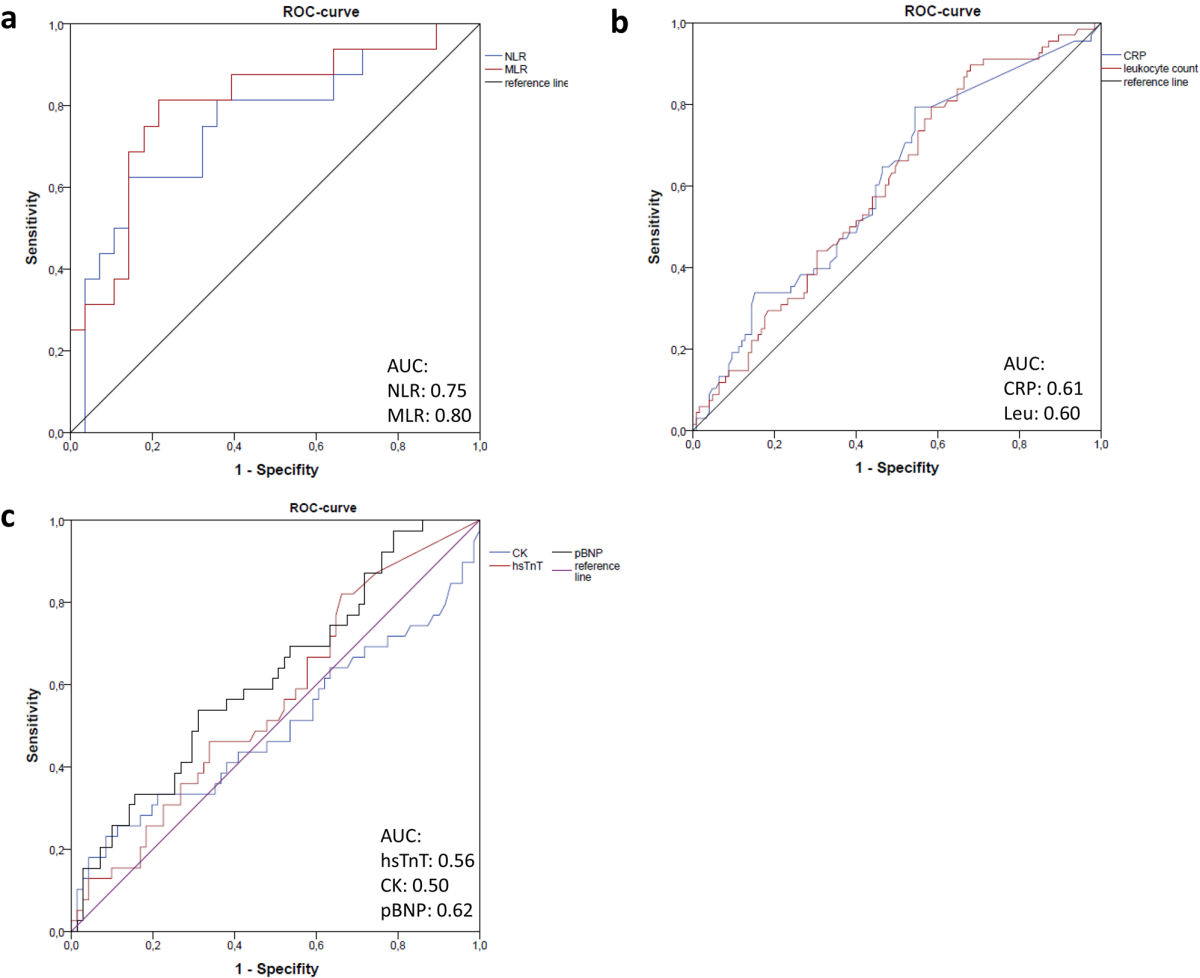
ROC-curves for prolonged hospital stay (≥ 7 days) of (**a**) NLR and MLR, (**b**) CRP and leukocyte count, (**c**) hsTnT, CK, pBNP. Abbreviations: NLR = neutrophil-to-lymphocyte ratio, MLR = monocyte-to-lymphocyte ratio, CRP = C-reactive protein, hsTnT = high sensitivity troponin T, CK = creatinine kinase, pBNP = pro brain natriuretic peptide.

ROC-analysis was conducted and AUC was calculated for the prediction of prolonged hospital stay (≥ 7 days; 75% percentile of data distribution) for NLR and MLR (NLR = 0.75, MLR = 0.80). Of note, AUCs for CRP, leukocyte count, hsTnT, creatinine kinase and pBNP were worse (CRP = 0.61, leukocyte count = 0.60, hsTnT = 0.56, CK = 0.50, pBNP = 0.62, see Fig. 2) for predicting prolonged hospital stay than AUCs of NLR and MLR.

Optimal cut-off for NLR and MLR were calculated by means of the Youden Index (NLR = 4.00 (sens.: 63%, spec.: 86%, PPV: 71%, NPV: 80%), MLR = 0.48 (sens.: 81%, spec.: 79%, PPV: 68%, NPV: 88%)). Of note, patients above the cut-off for NLR had a significantly higher prevalence of diabetes mellitus (28.6% vs. 3.4%, *p* = 0.032) and a non-significant trend towards higher prevalence of autoimmune disorders and active malignancies. A higher prevalence of diabetes mellitus was also observed in patients with an MLR above cut-off, however, this finding remained statistically insignificant (see Supplementary Table S2).

Of note, we did not find a statistically significant association of NLR, MLR, ELR or BLR with the combined endpoint of in-hospital complications or with all-cause-mortality after 2 years. However, our study was very likely underpowered in this regard (event rates: n = 25 and n = 3, respectively).

### MRI-subgroup

Additionally, we analyzed NLR and MLR only in the subgroup of patients who had evidence of myocarditis on MRI (n = 166, 83.4% of total cohort). Here, both NLR and MLR also showed a significant correlation with length of hospital stay (NLR: rs = 0.449, *p* = 0.005; MLR: rs = 0.524, *p* = 0.001), and a significant association with length of stay was also found in univariate linear regression analysis (NLR: β = 0.47, R^2^ = 0.23, *p* = 0.002; MLR: β = 0.53, R^2^ = 0.13, *p* = 0.016). AUC for prediction of prolonged stay was further calculated at NLR: 0.78 and MLR: 0.82 in this subgroup, which was again better than calculated AUCs for established biomarkers (see Supplementary Figure S1). However, there was still no association with the combined endpoint of in-hospital complications or all-cause-mortality after 2 years.

## Discussion

In the last two decades, several studies reported that neutrophil-to-lymphocyte ratio (NLR) and monocyte-to-lymphocyte ratio (MLR) correlate with the severity of different disease entities, such as sepsis, community-acquired pneumonia or coronary artery disease^8,9,12,15^.

In fact, neutrophils represent the first cellular line of defense of the innate immune response, and are soon recruited to oppose an impeding pathogen in case of an infection^20^. However, besides in the context of a response to infectious stimuli, peripheral neutrophil count is also affected by endogenous cortisol levels and the concentration of circulating catecholamines. As such, epinephrine and cortisol not only enlarge the pool of circulating neutrophils in blood, but also seem to extend the half-life of these cells, which results in neutrophilia^21–23^. Elevated levels of endogenous cortisol can furthermore result in lymphopenia, which can in part be explained by a redistribution of cells to lymphatic tissues^24–26^. Hence, NLR not only indicates infection or inflammation, but also represents a sensitive and rapid marker for physiologic stress^8^. In contrast, cortisol results in a depletion of circulating monocytes^27,28^, which is why the MLR predominately represents a marker of inflammation and infection^15^, and seems to be less affected by endogenous stress levels. Monocytes and macrophages also represent the majority of infiltrating cells in human and experimental myocarditis, and are thus crucial players in the pathophysiology of the disease^29^.

Because of the wide variation of clinical presentations, which range from paucisymptomatic forms to sudden cardiac death, myocarditis often poses a challenge to diagnostic workup in clinical practice. However, early identification and targeted treatment of patients at risk is crucial for the improvement of outcome^2,30,31^. Due to the promising results of previous studies, and the fact that NLR and MLR were not yet investigated in the context of myocarditis, we wanted to elucidate the predictive ability of these ratios in affected patients in our study.

In contrast to CRP and leukocyte count, which showed only slight elevations in our study cohort, NLR and MLR were markedly increased in patients with myocarditis (NLR: median 2.48 (IQR 1.55–4.58), normal value approx.: 1.77 ± 0.58^32,33^; MLR: median 0.42 (IQR 0.39–0.58), normal value approx.: 0.17 ± 0.05^32^).

Intriguingly, both NLR and MLR correlated with the length of hospital stay, and were better predictive for prolonged hospital stay (≥ 7 days) than established biomarkers like CRP, leukocyte count, high sensitivity troponin, creatinine kinase and pBNP. The calculated cut-offs therefor further showed adequate positive predictive values (PPV) and negative predictive values (NPV; NLR = PPV: 71%, NPV: 80%; MLR = PPV: 68%, NPV: 88%), which, together with the fact that the observed association with length of stay remained statistically significant even after correction for possible confounders in a multivariate linear regression model, further highlights the clinical implications of these two easily obtained ratios for everyday practice.

Furthermore, we observed a trend towards higher values of NLR in patients who suffered in-hospital complications (NLR: median 3.59 (IQR 1.60–10.13) vs. 2.35 (IQR 1.55–4.15), *p* = 0.211), which, however, remained statistically insignificant because our study was underpowered in this regard.

In line with the observation that NLR and MLR are associated with prolonged hospital stay, we found that the values of both ratios further correlated with clinical and laboratory parameters which can be interpreted as surrogates for the severity of myocarditis. As such, besides a significant correlation with CRP and leukocyte count, both ratios correlated positively with heart frequency and MLR further showed a significant inverse correlation with LV systolic function. Since MLR predominately represents a marker of inflammation^15^, and monocytes and macrophages are crucial players in the pathophysiology of myocarditis^29^, the latter finding could indicate that patients with a more pronounced inflammatory response had worse LV systolic function during hospitalization. However, the clinical implications of these correlations need to be investigated further in future studies.

Taken together, the findings of our study highlight that NLR and MLR could both be useful additional biomarkers in the risk stratification of patients with myocarditis, which is in line with previous studies on the predictive ability of these ratios^8–10,12,15^. In contrast, we did not find any relevant association of eosinophil-to-lymphocyte ratio (ELR) and basophil-to-lymphocyte ratio (BLR) with clinical, laboratory or outcome data in our study cohort.

## Conclusion

NLR and MLR showed a significant correlation with the length of hospital stay in patients with myocarditis, and were better predictive for prolonged hospital stay than established biomarkers like CRP, leukocyte count, high sensitivity troponin, creatinine kinase and pBNP. Furthermore, NLR and MLR correlated with clinical and laboratory parameters, which can be interpreted as surrogates for the severity of disease. Since both ratios are cost-efficient and rapidly available by obtaining white blood cell differentials, NLR and MLR could thus represent promising parameters in the risk stratification of patients with myocarditis, and could complement established biomarkers in the diagnosis and treatment of affected patients.

## Methods

Prior to enrollment, the study protocol was approved by the ethics committee of the state of Salzburg, Austria (EK Nr: 1181/2020). The study was conducted according to the principles of Good Clinical Practice and the Declaration of Helsinki.

### Patients

Eligible patients were identified through database search on discharge diagnoses of all patients admitted to the University Hospital of Salzburg, Austria, in the time-period of 2009 to 2019; discharge diagnoses were classified according to the International Classification of Diseases, Tenth Revision (ICD-10) diagnostic codes (I40.0, I40.1, I40.8, I40.9, I51.4). Database search identified 224 potential cases for study enrollment, which were further reviewed for eligibility by revision of all available hospital data records. A total of 202 patients were consecutively enrolled in the study. Patients were only enrolled if they fulfilled the diagnostic criteria for clinically suspected myocarditis, as defined by the European Society of Cardiology in its current position statement on myocarditis^34^. As such, patients were only included if they reported acute chest pain, had an abnormal 12-lead-ECG or regional wall motion abnormalities on transthoracic echocardiography (TTE), as well as elevated markers of myocardiocytolysis. Furthermore, patients were only included if they either had additional evidence of myocarditis on cardiac magnetic resonance imaging (MRI) or endomyocardial biopsy, or if other possible explanatory causes, such as coronary artery disease, hypertensive emergency or renal failure, had been excluded. Follow-up on all-cause-mortality was acquired for 2 years after presentation.

### Endpoints

The primary endpoint was length of hospital stay due to myocarditis.

Secondary endpoints were a combined endpoint of in-hospital complications (in-hospital-mortality, relevant arrhythmias (i.e. arrhythmias requiring therapeutical intervention, such as ventricular/supraventricular tachyarrhythmia or bradyarrhythmia), cardiogenic shock, necessity for mechanical ventilation) and all-cause-mortality after 2 years.

### Statistical analysis

Statistical analyses were conducted with R (version 4.0.2., R Core Team (2013), R Foundation for Statistical Computing, Vienna, Austria; http://www.R-project.org/) with the packages ‘ggplot2’, ‘glmnet’, ‘pastecs’, ‘Hmisc’, ‘ggm’ and ‘QuantPsyc’. Distribution of data, skew and kurtosis were assessed visually and by Shapiro–Wilk test. Because data distribution not normal, continuous data are depicted as median ± interquartile‐range (IQR). Medians were compared using by Mann–Whitney-U Test. Multiple linear regression analysis was conducted using the stepwise method with backward direction, missing variables were excluded from the analysis. Durbin Watson test was conducted to check for autocorrelation, Cook’s distance and leverage were calculated to check for influential cases. Variance inflation factors are depicted in Table 4. ROC analysis was performed and AUC was calculated for NLR and MLR. Optimal cut‐offs were calculated by means of the Youden Index^35^. A *p* value of < 0.05 was considered statistically significant.

### Ethics declaration

The study was conducted according to the Declaration of Helsinki and the principles of Good Clinical Practice. The study protocol was reviewed and approved by the ethical review board of the state of Salzburg, Austria (EK Nr: 1181/2020) prior to patient enrollment. The need to obtain informed consent from patients was waived for by the ethical review board of the state of Salzburg, Austria, since it was a retrospective observational study only.

### Limitations

Our study has several limitations. First, our study was conducted at a single study center only and the study design was retrospective. Due to the lower level of evidence, a retrospective design is inferior to a prospective study design, however, because of the low incidence of myocarditis, we chose this design to test our hypotheses first; another prospective trial is being planned by our study group to confirm our current findings.

Second, since we wanted to investigate a real-world cohort of patients with myocarditis, we chose to include patients according to the diagnostic criteria for clinically suspected myocarditis by the ESC^34^. However, this resulted in patients being enrolled in the study who did not have an MRI or endomyocardial biopsy (EMB) to prove myocarditis. In our practical experience, cardiac MRI and EMB are sometimes not conducted if the clinical presentation speaks highly in favor of myocarditis and if other causes of an elevation of cardiac enzymes, such as coronary artery disease, hypertensive emergency or renal failure, have been ruled out. Hence, all of the patients enrolled in this study either additionally had evidence of myocarditis on MRI or EMB, or coronary angiography, coronary computed tomography or myocardial szintigraphy had been conducted to rule out coronary artery disease. All cases were further checked for presence of other explanatory causes for an elevation of cardiac enzymes and, if applicable, excluded from the study.

Furthermore, we did not find a statistical association of NLR, MLR, ELR and BLR with all-cause-mortality, the necessity for mechanical ventilation, the presence of arrhythmias or cardiogenic shock. However, our study was very likely underpowered in this regard- future multi-center studies should address this issue further. Lastly, since length of hospital stay is merely a surrogate parameter for the severity of a disease (i.e. patients with more severe disease are likely to require longer hospital treatment than more healthy patients), several other parameters, such as additional diagnostic procedures or treatments arising from other comorbidities, can bias the length of stay. This should be considered when interpreting the results of our study.

## Supplementary Information

Supplementary Information.

## Data Availability

The data underlying this article will be shared on reasonable request to the corresponding author.
