# Supplementary material for: Neutrophil-to-lymphocyte ratio and monocyte-to-lymphocyte ratio predict length of hospital stay in myocarditis
Source: Sci Rep. 2021 Sep 13;11:18101. doi: 10.1038/s41598-021-97678-6 (PMC8438016; doi:10.1038/s41598-021-97678-6)
Supplement: Supplementary file 1 — Supplementary Information. [file 41598_2021_97678_MOESM1_ESM.pdf]

Neutrophil-to-lymphocyte ratio and monocyte-to-lymphocyte ratio predict length of hospital stay in myocarditis.

-Supplementary materials-

Moritz Mirna[1*], MD, PhD; Lukas Schmutzler[1*]; Albert Topf[1], MD; Prof. Uta C. Hoppe[1], MD; Assoc.Prof. Michael Lichtenauer[1], MD, PhD

[1] Department of Internal Medicine II, Division of Cardiology, Paracelsus Medical University of Salzburg, Austria

*contributed equally

Correspondence:

Moritz Mirna, MD, PhD

Department of Internal Medicine II
Division of Cardiology
Universitätsklinikum der Paracelsus Medizinischen Universität
Müllner Hauptstraße 48
5020 Salzburg
Austria

e-Mail: m.mirna@salk.at

Telephone: +43 (0) 57255 - 58340

ORCID iD: https://orcid.org/0000-0001-5679-4872

Supplementary Figure S1

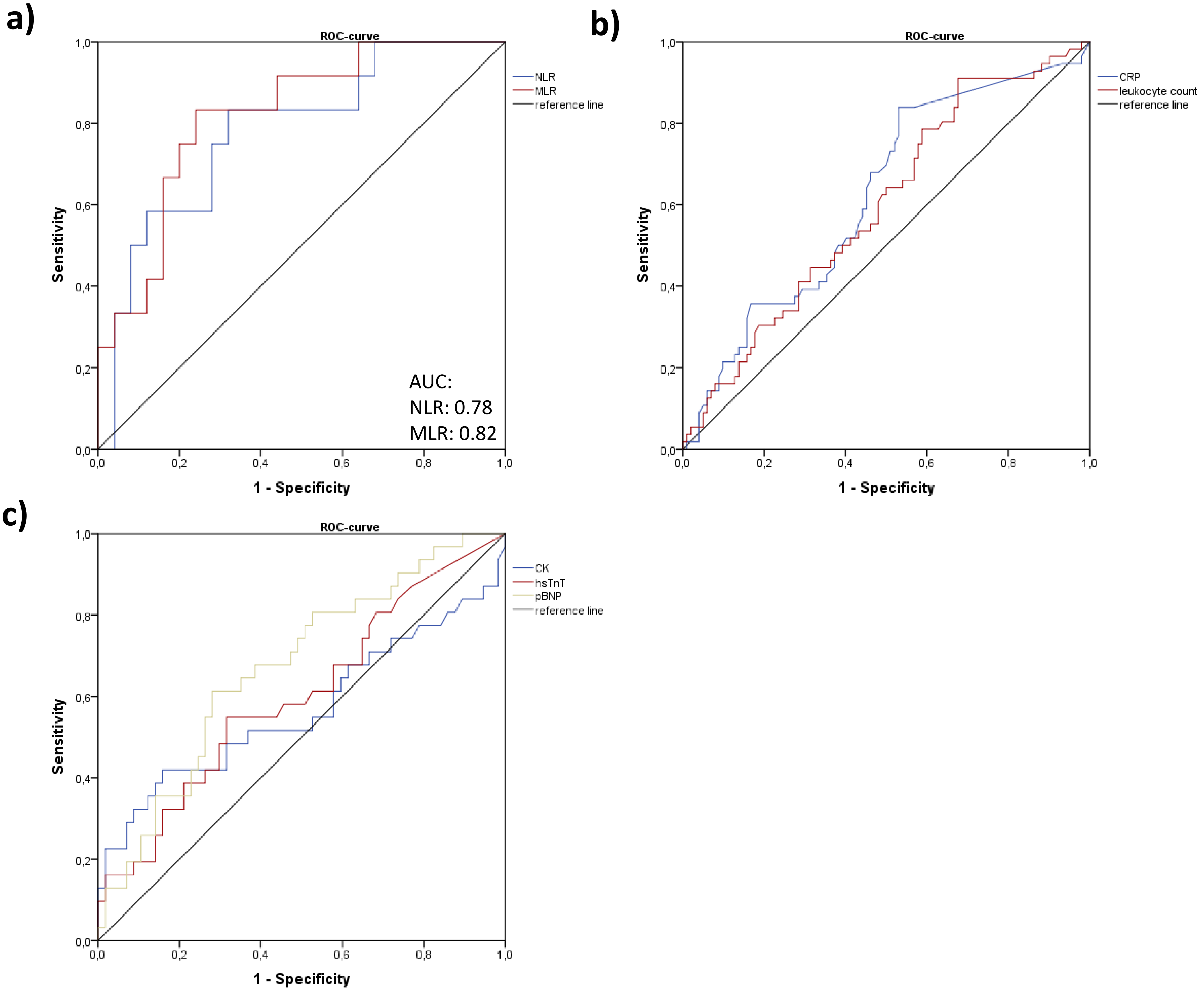

Suppl. Fig. S1: ROC-curves for prolonged hospital stay (≥ 7days) in the subgroup of patients only with evidence of myocarditis on MRI (n= 166, 83.4% of cohort) of a) NLR and MLR, b) CRP and leukocyte count, c) hsTnT, CK, pBNP. *Abbreviations: NLR= neutrophil-to-lymphocyte ratio, MLR= monocyte-to-lymphocyte ratio, CRP= C-reactive protein, hsTnT= high sensitivity troponin T, CK= creatinine kinase, pBNP= pro brain natriuretic peptide.*

dependent variable: length of stay<br><br><u>adjustment for:</u> diabetes mellitus, obesity, active smoking status, autoimmune disorder, chronic infectious disease, active malignancy					
	**B**	**std. error**	**95%CI**	**β**	**p-value**
**NLR**	0.382	0.151	0.073-0.690	0.179	0.017
**MLR**	7.238	2.356	2.419-12.056	0.236	0.005

Suppl. Table S1: Linear regression analysis for length of hospital stay with adjustment for possible confounders. Variance inflation factors (VIF): diabetes mellitus: 1.093, obesity:1.046, active smoker: 1.037, autoimmune disorder: 2.618, immunosuppression: 2.475, active malignancy: 1.366, chronic infectious disease: NA. *Abbreviations: NLR= neutrophil-to-lymphocyte ratio, MLR= monocyte-to-lymphocyte ratio.*

	NLR < 4.00	NLR ≥ 4.00			MLR < 0.48	MLR ≥ 0.48	
	median (IQR)	median (IQR)	p-value		median (IQR)	median (IQR)	p-value
**Length of Stay (days)**	4 (3-6)	9 (6-12)	0.001		4 (3-5)	8 (5-10)	<0.0001
**Age (years)**	32 (24-44)	48 (28-62)	0.057		37 (25-45)	42 (24-50)	0.499
**Systolic blood pressure (mmHg)**	135 (118-150)	122 (115-164)	0.990		130 (117-143)	150 (120-157)	0.432
**Diastolic blood pressure (mmHg)**	84 (69-90)	70 (64-89)	0.500		80 (66-90)	84 (65-90)	0.967
**Heart frequency (bpm)**	74 (60-81)	88 (69-105)	0.034		74 (59-81)	83 (67-90)	0.083
**Peripheral oxygen saturation (%)**	98 (97-99)	95 (92-97)	0.250		98 (96-98)	96 (92-98)	0.857
**Temperature (°C)**	36.8 (36.2-37.2)	36.1 (35.5-39.5)	0.769		36.8 (36.1-37.0)	36.4 (35.8-38.7)	0.898
	% (n)	% (n)	p-value		% (n)	% (n)	p-value
**Male sex**	73.3 (22)	64.3 (9)	0.724		68.0 (17)	73.7 (14)	0.749
**Diabetes mellitus**	3.4 (1)	28.6 (4)	0.032		4.2 (1)	21.1 (4)	0.153
**Hyperlipidemia**	13.8 (4)	14.3 (2)	0.965		12.5 (3)	15.8 (3)	0.757
**Obesity (BMI >30kg/m²)**	20.0 (6)	7.1 (1)	0.401		24.0 (6)	5.3 (1)	0.119
**Arterial hypertension**	10.3 (3)	28.6 (4)	0.190		12.5 (3)	21.1 (4)	0.680
**History of smoking**	27.6 (8)	28.6 (4)	0.946		16.7 (4)	42.1 (8)	0.091
**Chronic infectious disease**	0 (0)	0 (0)	NA		0 (0)	0 (0)	NA
**Autoimmune disorder**	6.7 (2)	14.3 (2)	0.581		8.0 (2)	10.5 (2)	0.773
**Immunosuppressive therapy**	6.7 (2)	7.1 (1)	0.953		8.0 (2)	5.3 (1)	0.721
**Active malignancy**	3.3 (1)	21.4 (3)	0.088		8.0 (2)	10.5 (2)	0.773

Suppl. Table S2: Baseline characteristics and comorbidities between patients above and below the calculated cut-offs for NLR and MLR. *Abbreviations: NLR= neutrophil-to-lymphocyte ratio, MLR= monocyte-to-lymphocyte ratio.*
